# A Controlled Before-and-After Perspective on the Improving Maternal, Neonatal, and Child Survival Program in Rural Bangladesh: An Impact Analysis

**DOI:** 10.1371/journal.pone.0161647

**Published:** 2016-09-01

**Authors:** Mahfuzar Rahman, Fakir Md. Yunus, Rasheduzzaman Shah, Fatema Tuz Jhohura, Sabuj Kanti Mistry, Tasmeen Quayyum, Bachera Aktar, Kaosar Afsana

**Affiliations:** 1 Research and Evaluation Division, BRAC, BRAC Centre, 75 Mohakhali, Dhaka, Bangladesh; 2 Department of Global Health, Save the Children USA, Washington, DC, United States of America; 3 Health, Nutrition, and Population Programme, BRAC, BRAC Centre, 75 Mohakhali, Dhaka, Bangladesh; 4 James P Grant School of Public Health, BRAC University, 68 Shahid Tajuddin Ahmed Sharani, Mohakhali, Dhaka, Bangladesh; Stellenbosch University, SOUTH AFRICA

## Abstract

**Objectives:**

We evaluated the impact of the Improving Maternal, Neonatal, and Child Survival (IMNCS) project, which is being implemented by BRAC in rural communities in Bangladesh.

**Methods:**

Four districts received program intervention i.e. trained community health workers to deliver essential maternal, neonatal, and child healthcare and nutrition services while two districts were treated as comparison group. A quasi-experimental study design (compared before-and-after) was undertaken. Baseline survey was conducted in 2008 among 7200 women followed by end line in 2012 among 4800 women with similar characteristics in the same villages. We evaluated maternal antenatal and post natal checkup, birth plans and delivery, complication and referred cases during antenatal checkup and post natal period, and child health indicators such as birth asphyxia, neonatal sepsis, and its management by the medically trained provider.

**Findings:**

Increased number (four or more) antenatal visits, skill-birth attended delivery and postnatal visits (three or more) in the intervention group preceding four-year intervention period were observed compare to their counterpart. We noted negative difference-in-difference estimator (-5.0%, P = 0.159) regarding to the all major birth plans i.e. delivery place, birth attendant, and saved money in the comparison areas. Significant reduction of ante-partum and intra-partum complications occurred in the intervention group, contrary complications of such event increased in the comparison areas (-6.3%, P<0.05 and -20.5%, P<0.001 respectively). Referral case to the health centers due to these complications boosted significantly in intervention group than comparison group (2.3%, P<0.01 and 6.6%, P<0.001 respectively). Mother’s knowledge of breastfeeding initiation and the practice of initiating breastfeeding within an hour of birth amplified significantly (14.6%, P<0.001 and 8.3%, P<0.001 respectively). We did not find any significant difference regards to the management of low birth weight by the medically trained health care provider and complete vaccination between the intervention and comparison arm.

**Conclusion:**

Medically trained health care provider assisted community based public health intervention could increase number of antenatal and postnatal visit, thereby could decrease pregnancy associated complications. These interventions may be considered for further up scaling when resources are limited.

## Introduction

There has been substantial progress in reducing maternal, neonatal and under-five mortality rates over the past two decades [1]. Between 1990 and 2013, the global maternal mortality ratio (MMR) decreased by 45% (380 to 210 deaths per 100,000 live births) [2].Similarly, the global neonatal mortality rate (NMR) decreased by 30% (33 to 21 deaths per 1,000 live births)and under-five mortality by 41% (90 to 48deaths per 1,000 live births) over the same period [1, 3].Despite these successes, current rates of decline in maternal, neonatal, and under-five mortality rates will not reach the global targets of a three-quarter reduction in maternal mortality and a two-thirds reduction in under-five child mortality by 2015 [4]. Furthermore, inequalities in access to health services widen the gap in mortality rates between the rich and poor in low-income countries (LICs), which account for 99% of global maternal deaths [5]; the MMR is reported to be14-times higher in LICs than developed countries [4]. Specifically, sub-Saharan Africa has 510 maternal deaths per 100,000 live births and South Asiahas190 maternal deaths per 100,000 live births [6–8].

There are significant disparities in the urban-rural utilization of maternal health services in Bangladesh, creating challenges to improving maternal health. According to the Bangladesh Demographic and Health Survey 2014 (BDHS 2014), medically trained personnel attend only42% of births (urban 48.9% vs. rural 24.9%). Furthermore, only 34% of women receive postnatal care (PNC) from a medically-trained provider within 48 hours of delivery [9]. By following global community advice, the Government of Bangladesh (GoB) has set its own goal to improve maternal and child health as per the Millennium Development Goals (MDGs), particularly MDG 4 (reduce child mortality rate) and MDG 5 (improve maternal health) by 2015. To keep pace with MDG targets and GoB strategies, different governmental (GO) and non-governmental organizations (NGO), bilateral agencies, and donors have implemented various health interventions either individually or in partnership with the government to achieve MDG 4 and 5 targets, particularly amongst the poor.

The definite positive impact of maternal and neonatal health outcomes [10–14] necessitates evaluation of methods that might further improve outcomes. For example, it is widely advocated that communities should be actively involved in improving their own health. Specifically, grassroots-level health workers belonging to the community are ideally placed to intervene in that community. In Kenya, community engagement and service-strengthening interventions have improved family planning practices [15], and they are now considered effective tools for mobilizing the community. However, direct evidence of the effects of community participation on specific health outcomes is limited. We hypothesized that a large number of community health workers with an effective referral system could potentially improve maternal health services. This study aimed to evaluate the potential of community mobilization using frontline workers as an approach to improving a range of health-related outcomes in mothers, neonates, and children less than five years of age in rural Bangladesh.

## Methods

### Study design, setting and population

A quasi-experimental study design (non-randomized assignment of the intervention and comparison group) was used to measure IMNCS project impact on maternal, neonatal, and child health (MNCH) outcomes over a period of five years (2008 to 2012) between the intervention and comparison group among the six districts of Bangladesh [16]. Among them, four districts (Nilphamari, Gaibandha, Rangpur, and Mymensingh) were considered as intervention areas who received the IMNHS intervention and two were designated comparison districts (Naogaon and Netrakona) who did not received IMNCS intervention. Both comparison districts are situated in the north pole of Bangladesh (geographically close to the intervention districts) and share same socio-demographics characteristics. During the period ofIMNCS intervention, BRAC was active in all six study districts supported by the core programs in microfinance, health, and education. Typical activities included providing beneficiaries with education and skill development training, social awareness campaigns, and microfinance programs to support income-generating activities.

**Fig 1:**
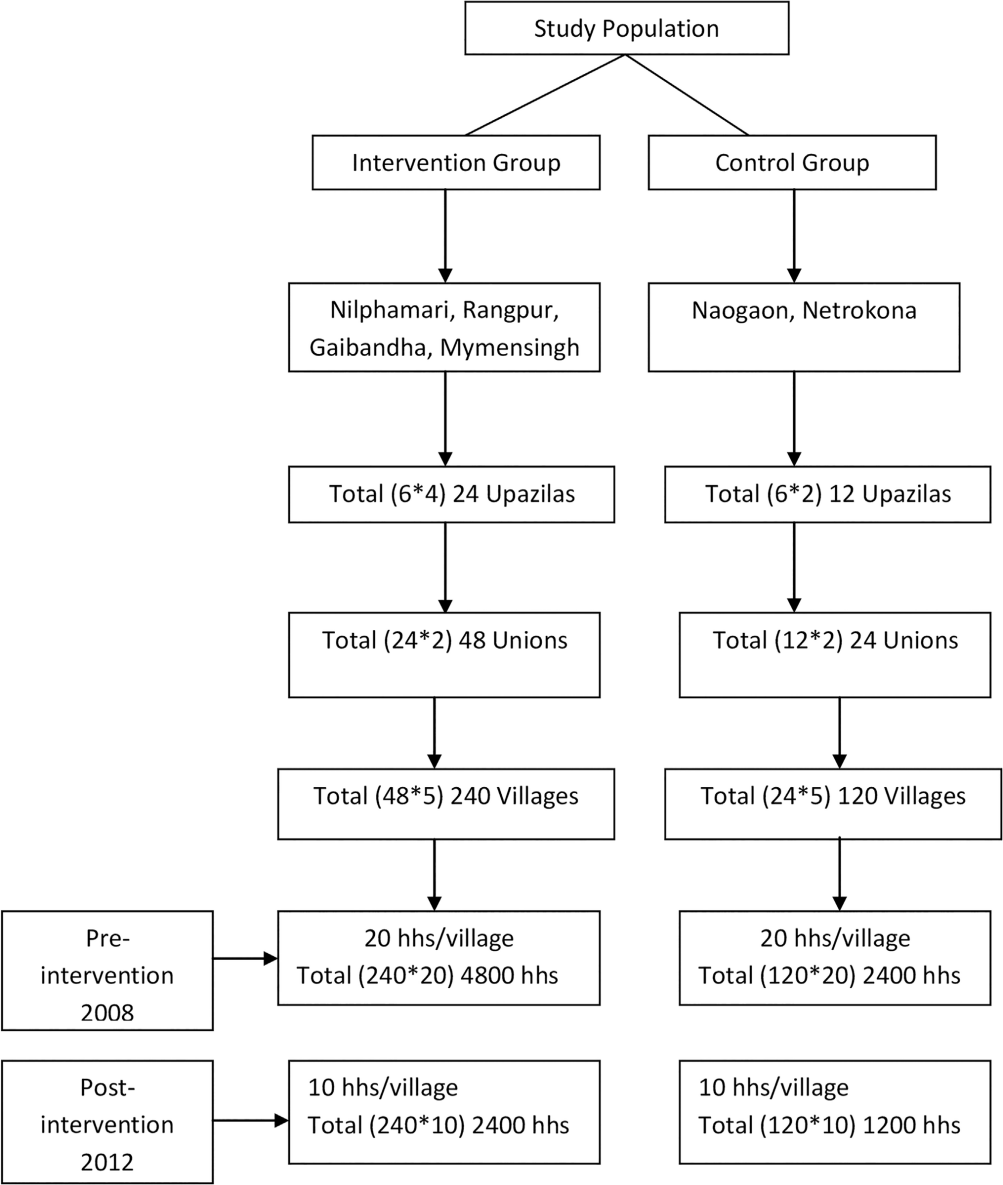
Study population.

Multi-stage random sampling was used to select the representative respondents from each district. Survey participants were randomly selected from all eligible mothers, i.e. those who had a living child aged less than one year or had given birth to a child who had died, a pregnancy terminated, an intrauterine death (IUD), or still birth in the previous year and whose youngest child was aged 12–59 months. In 2008, the estimated sample size was 4,800 and 2400 eligible mothers in intervention and comparison areas were interviewed respectively (1200 per district), based on the rates of skilled delivery with a health worker and prevalence of antenatal and postnatal care etc. The sample was calculated based on80% power to detect a change of 50% (with 5% error level) and a design effect of 1.5. A non-response rate of3% was assumed. The same sampling strategy was used in 2012, assuming an expected change in prevalence due to IMNCS intervention and the resulting sample was 4,800 and 2400 eligible mothers in intervention and comparison areas were interviewed respectively (600 per district, see Fig 1).

### Overview of the program intervention: The Improving Maternal, Neonatal and Child Survival (IMNCS) Program

The IMNCS Program initiated by BRAC, the largest NGO in the world, has nearly 40 years’ experience in implementing community-based health interventions in both rural and urban settings via the BRAC Health, Nutrition, and Population Programmed (HNPP). At the end of 2005, BRAC launched a comprehensive maternal, neonatal, and child health (MNCH) initiative in Nilphamari, a poverty-stricken district of northern Bangladesh, to develop a model MNCH intervention. Inspired by the lesson learned and after rigorous evaluation of the project design and output, program coverage has been extended in phases to more districts since 2008. In 2014, the project was implemented in a total of 14 districts. Prior to 2012, the project was a collaborative effort between BRAC, UNICEF, and the Directorate General of Health Services (DGHS) of the Government of Bangladesh (GoB) in an effort to accelerate progress toward MDG 4 and MDG 5 targets. After 2012, BRAC and the DGHS of GoB continued the collaboration to deliver a project to reduce maternal, neonatal, and child mortality and morbidity, particularly among poor and socially excluded populations in rural Bangladesh. Specifically, the main objectives were: 1) to increase knowledge and improve practices related to maternal, neonatal, and child health; 2) to improve provision of quality maternal, neonatal, and child health services at the household and community levels; 3) to increase availability and access to a quality continuum of maternal, neonatal, and child healthcare and services at facilities; 4) to increase informed demand for, and utilization of, community, facility, and referral services; and 5) to increase participation, accountability, and responsiveness to communities’ voices in maternal, neonatal, and child health services. Women were eligible to receive this intervention from the very start of their pregnancy until their child was five years old. The intervention components used in intervention and comparison districts are detailed in our earlier publication [16] and Table 1.

**Table 1:** Intervention package description.

	Intervention (Services provided by program)	Comparison (Services provided by EHC)
Formation of village-based maternal neonatal child health (MNCH) committees	√	×
Training of traditional birth attendants (TBAs) on safe deliveries and ties with community health workers (CHWs)	√	×
Promotion of antenatal and postnatal care practices	√	√
Promote tetanus toxoid during pregnancy	√	√
Promotion of birth planning	√	×
CHWs’ attendance at delivery	√	×
Counseling and communication strategies	√	√
Effectivereferralsystem^1^	√	×
Knowledge provided by CHWs to mothers about maternal and neonatal danger signs	√	×
Promotion of adequate maternal nutrition and rest	√	√
Immediate newborn care including cord care	√	×
Promotion of exclusive breastfeeding	√	×
Ensuring colostrum feeding and early initiation of breastfeeding (within 1 hour of birth)	√	×
Age-specific complementary feeding	√	×
Promotion of delayed bathing (after 6 hours of delivery)	√	√
Formation of village-based MNCH committees	√	×
Training of TBAs on safe deliveries and ties with CHWs	√	×
Promotion of antenatal and postnatal care practices	√	√

^1^Effective referral system = prompt referral, arranged transport, facility management.

In our previous article we assessed IMNCS program descriptively [16]and in this paper we aimed to measure the impact of the IMNCS program implemented by BRAC HNPP.

### Intervention vs. comparison group

The IMNCS intervention included intensive maternal and newborn care, in contrast to the BRAC EHC. Larger numbers of Shastho Shebikas (community health worker) were involved in the IMNCS areas compared to the comparison areas, which, along with effective referral, was assumed to improve maternal health services, e.g., number of antenatal care visits, and antenatal and delivery care delivered by trained providers. They are the nucleus of the BRAC HNPP who is basically a community health worker (age ≥ 25 years, and married having children not below two years) selected from same village where they will be working on. In a typical month, each of the Shastho Shebika visits around 200–250 households (usually visits 15 households daily) to disseminate universal health and nutrition messages such as family planning and immunization, motivate for installing sanitary latrines, provide pregnancy-related care and treatment for some common illnesses, identify TB suspects and sells health commodities. After selection, all Shastho Shebikas and Shastho Kormis (BRAC HNPP salaried staff who supervise Shastho Shebikas) underwent extensive residential training on maternal, newborn, and child health issues. A group of doctors and general trainers were involved in developing training materials and conducting the process under continuous guidance from the central BRAC’s HNPP training cell. These trained community health workers (Shastho Shebikas and Shastho Kormis) identified pregnancies in the intervention areas and provided domiciliary maternal health services such as antenatal care, post natal care, delivery care, essential newborn care, under five care for acute respiratory infection and diarrhea, and education on maternal, neonatal, and child health and nutrition issues in the community. The Shastho Shebikas referred the mothers and children to health facilities when complications arose and often accompanied pregnant mothers referred to facilities in intervention areas. The comparison group received only BRAC Essential Health Care program including health and nutrition education, water and sanitation, family planning, immunization, pregnancy-related care, vitamin A supplementation, and basic curative services; no intensive maternal care and referral support was provided. The services were provided by the Shastho Shebikas of the essential health care program. The health services delivered via IMNCS in the intervention districts and BRAC EHC programme in the comparison districts are summarized in Table 1.

### Outcome variables

Outcome variables were categorized and separately analyzed by maternal and infant and child health indicators. Among the maternal health outcome, we further sub-categorize into women who have any outcome in previous one year or have under five children, mothers who have any outcome last one year, and mothers with under-1 live children or whose under-1child died in previous year. Information related to women’s pregnancy status and use of family planning methods were placed under the women who have any outcome in previous one year or have under five children. Mother’s antenatal checkup history by the trained health care provider, birth plans and delivery, complication and referred cases during antenatal checkup and after delivery were considered under the mothers who have any outcome last one year. History of postnatal care, complication and referred cases were placed under the mothers with under-1 live children or whose under-1child died in previous year subcategory. Regards to the infant and child health outcome indicators, two sub-categories were created. Knowledge on the newborn, low birth weight, breast feed initiation just after birth (within 1 hour), and prevalence of neonatal morbidity such as birth asphyxia, neonatal sepsis, and its management by the medically trained provider were considered under the mothers with live children less than one-year-old or whose child less than one died in past year subcategory. In the second subcategory, vaccination, acute respiratory infection (ARI), diarrhea, and other illness were placed under the women who have live child.

### Data collection tools and techniques

The baseline (2008) and follow-up (2012) surveys were conducted using structured questionnaires. The Bangladesh Health and Demographic Survey (BDHS) questionnaire was used to collect data on the socio-demographic characteristics of respondents including their age, education, occupation, religion, non-government organization involvement, and husband’s socio-demographic characteristics. The survey also collected information on household characteristics including household assets, sanitation and drinking water accessibility, and materials used for the floor, roof, and walls. Questions regarding reproductive history included age at marriage and conception, number of children, previous child deaths, and information on the use of family planning methods. Maternity care information included questions on antenatal care, delivery, and postnatal care and on complications and treatment-seeking behavior during the antenatal, delivery, and postnatal period. Information on treatment-seeking behavior for newborns with respect to vaccinations and childhood illnesses was also collected. Information on ante-partum, peri-partum, post-partum, and newborn care was obtained from mothers belonging to the group with a pregnancy outcome in the previous12 months (group 1); mothers who had received terminations or experienced intrauterine deaths (IUDs) or infant death were not asked about neonatal or child care. Mothers of children aged 12–59 months (group 2) were asked about child immunization, vitamin A intake, and their experience of under-fives illnesses. Both the 2008 and 2012 survey questionnaires were pre-tested and revised based on feedback received during field testing. Seventy enumerators and six monitors were recruited for household listing and surveys. Enumerators of both surveys received ten days of training consisting of lectures and activities to improve interviewing skills. At the end of training, participants spent two days in the field conducting a pilot survey followed by review and evaluation. Data were collected during October to January in 2008 and 2012.

### Co-variables

Data were collected on the participants’ socio-demographic characteristics, reproductive and family planning histories in two categories: pregnant mothers and related to newborn care. Data on maternity care were captured and included details of antenatal care, delivery, and postnatal care. We also gathered data on postnatal complications and treatment-seeking behavior during antenatal care, delivery, and postnatal care. Detailed newborn histories were taken and included information on newborn care, complications, and treatment-seeking behavior.

### Statistical analysis

The Difference-in-Difference (DID) estimator was used to the panel sample of baseline and endline observations to estimate the effect of intervention “exposed” individuals relative to comparison “unexposed” individuals. The measure of the causal effect is represented by the coefficient on the interaction term β_3_ in a Ordinary Least Squares (OLS) regression of the outcome of interest Y_*it*_ (e.g. currently using modern FP method or not, received at least four ANCs from trained provider) for the individual mother *i* by the time *t* (the two period) Eq (1). The procedure we used for the Difference-in-Difference regressions follow as below:

$$Y_{it}=\beta_{0}+\beta_{1}\chi_{it}+\beta_{2}S_{i}+\beta_{3}{Τ}_{12}+\beta_{4}S_{i}*{Τ}_{12}+\varepsilon_{it}$$ (1)

Where X_it_ represents a set of control variables that could also impact health outcomes including; Age, literacy of respondent and her husband, years of completed schooling of respondent and her husband, wealth status, involvement in income generating activities, NGO involvement etc. *S* is a binary variable that equals 1 if individual *i* was in an area that received health intervention (treated individuals), and 0, otherwise[17]. We tested whether individuals located within the areas where health intervention was provided to benefit from intervention. *T*_12_ is a dummy that equals 1 if year *t* is 2012, and 0, otherwise. Accordingly, 2008 was our baseline year, and the end-line was 2012. We estimate this model Eq (1) at the individual level. The main parameter of interest is *β*_4_, which the Difference-in-Difference estimator is and it is an indicator of whether intervention recipient individuals fared better or worse than the untreated individuals (i.e. comparison group) between the two time periods of 2008 and 2012. This estimator is computed after controlling for original individual and village characteristics. Analyses were conducted using STATA Version 12 statistical software.

### Ethical approval

The ethical approval was obtained from the Bangladesh Medical Research Council (BMRC). The objective including the risk and benefit of the study was comprehensively explained to each participant. Informed verbal consent was obtained from study participants in the presence of a witness for their information to be stored in Research and Evaluation Division, BRAC. Since each of the respondents agreed to participate voluntarily, interviewers were then put a tick mark in the ‘I agree to participate’ box in the consent form in front of a witness and documented accordingly. The respondents were ensured of the confidentiality of the information provided and informed that their provided information will be used only for research purpose. We accepted informed verbal consent from the respondents because from our previous experiences, most participants are not comfortable with the idea of signing/inserting thumbprint in the consent form as generally people consider signature or thumbprint as a legal document of transferring property. We obtained information and taken informed verbal consent from the kin, caretakers, or guardians in case of children. The study and consent procedures were approved by the Ethical Committee of the Bangladesh Medical Research Council."

## Results

The socio-demographic characteristics of the respondents are summarized in Table 2. Literacy rates and educational status significantly improved in both the intervention and comparison districts between 2008 and 2012. Although the majority of respondents’ husbands had no literacy, secondary educational status increased in both the intervention and comparison group. More than 50 decimal of land holding decreased significantly in both the intervention and comparison districts, but more in the comparison districts. A greater percentage of respondents own less than 50 decimal land, but there was significant variation in both groups before and after intervention (P<0.001).

**Table 2:** Respondents’ socioeconomic characteristics before and after the intervention.

	Intervention			Control		
	2008	2012	P-value	2008	2012	P-value
Mother of under-1 live Child	1995	974		984	485	
Mother whose under-1 Child died in the past year	97	49		42	19	
Mother who had termination or MR or stillbirth or IUD in past year	308	177		174	96	
Mother of children aged 12–59 months	2400	1200		1200	600	
Total respondents	4800	2400		2400	1200	
Respondent’s age (years) (%)						
≤19	14.7	18.5	0.000	14.8	16.2	0.54
20–34	76.7	75.2		76.8	75.8	
≥35	8.6	6.4		8.4	8.0	
Mean maternal age	25.39	24.78		25.23	25.09	
Respondent’s literacy (%)						
Yes	50.0	58.5	0.000	52.4	61.9	0.000
No	50.0	41.5		47.6	38.1	
Respondent’s education (%)						
No education	34.6	19.2	0.000	33.9	23.0	0.000
Primary education (1–5)	29.1	34.7		32.0	33.9	
Secondary education (6–10)	29.4	36.9		28.5	35.2	
Higher secondary	6.9	9.2		5.6	7.9	
Respondent’s involved in income earning (%)						
Yes	15.4	6.9	0.000	11.5	12.8	0.000
No	84.6	93.1		88.5	87.2	
Husband’s literacy (%)						
Yes	44.2	50.9	0.000	45.3	51.8	0.000
No	55.8	49.1		54.7	48.2	
Husband’s education (%)						
No education	46.4	33.3	0.000	45.8	36.2	0.000
Primary education (1–5)	24.0	31.2		24.2	29.4	
Secondary education (6–10)	16.8	19.5		18.3	21.8	
Higher secondary	12.1	14.7		11.1	11.9	
Amount of land (%)						
None	4.8	24.2	0.000	3.2	14.9	0.000
< = 50 dcm	64.8	51.2		56.2	54.1	
>50 dcm	30.4	24.6		40.6	31.0	
Wealth index (%)						
Lowest	19.2	15.7	0.000	24.7	22.5	0.000
Second	20.2	22.7		16.3	21.0	
Middle	18.9	23.9		17.2	21.9	
Fourth	19.8	20.2		21.2	18.6	
Highest	21.9	17.6		20.6	16.0	

The difference-in-difference analyses for key maternal health indicators are presented in Table 3 (also S1 Table). The effects of intervention on antenatal care, deliveries attended by community-based skilled birth attendants, and postnatal care were especially high in the four-year exposure area compared to the comparison area. Results show that the proportion of women using modern family planning methods significantly increased (5.3%, p<0.05). There was a much larger increase in the proportion of women who received four or more antenatal care visits from trained providers than in the comparison group in the preceding four-year intervention period, with a similar result seen for the proportions experiencing three or more postnatal care visits (31.4%, P<0.001 and 16.7%, P<0.001, respectively). The majority of women in comparison groups had all major birth plans (delivery place, birth attendant, and saved money), resulting in a negative difference-in-difference estimator (-5.0%, P = 0.159), although this was not significant. Complications during the ante-partum and intra-partum period were decreased in intervention group but it increased higher in comparison group (-6.3%, P<0.05 and -20.5%, P<0.001 respectively), simultaneously the proportion of women referred due to these complications increased significantly in intervention group than comparison group (2.3%, P<0.01 and 6.6%, P<0.001 respectively). Although the rate of increase was higher in intervention group, no significant differences were observed in deliveries assisted by trained providers. The proportion of women and their families having all major birth plan decreased in the intervention districts over time by 5%.

**Table 3:** DID analysis of the impact of the IMNCS intervention on maternal health indicators.

Outcomes	Intervention		Comparison		DID estimate	95% CI	P value
	Pre	Post	Pre	Post			
**Women who have any outcome last one year or have under five child (n)**	**4800**	**2400**	**2400**	**1200**			
Women first conceive before age 19 (%)	69.5	73.5	68.5	68.5	3.0	-0.9 to 6.8	0.137
Currently using modern FP method (%)	57.0	64.1	59.8	62.2	5.3	1.0 to 9.7	0.016**
**Mother who have any outcome last one year (n)**	**2400**	**1200**	**1200**	**600**			
Received at least four ANCs from trained provider^1^(%)	24.1	69.2	15.5	28.2	31.4	24.4 to 38.3	0.000***
Had all major birth plans (place, attendant and saved money) (%)	46.6	39.9	38.1	35.8	-5.0	-11.9 to 1.9	0.159
Delivered by trained birth attendant^1^ (%)	33.7	55.0	37.3	54.7	2.6	-3.8 to 8.9	0.423
Complication faced during antenatal period (%)	14.8	12.2	17.5	21.3	-6.3	-11.4 to -1.2	0.016**
Complication faced during delivery period (%)	20.5	17.8	24.5	33.8	-12.6	-18.9 to -6.2	0.000***
Referred for antenatal complications (%)	1.8	3.7	1.4	1.0	2.3	0.6 to 4.0	0.008***
Referred for delivery complications (%)	1.9	10.0	1.0	2.3	6.6	4.2 to 9.2	0.000***
**Mother with under-1 live children or whose under-1child died in past year (n)**	**2092**	**1023**	**1026**	**504**			
Received at least three PNC (%)	11.8	37.6	10.0	18.1	16.7	10.9 to 22.5	0.000***
Complication faced during postnatal period (%)	12.0	8.9	15.8	10.9	1.4	-3.3 to 6.1	0.551
Referred for postnatal complications (%)	0.4	2.0	0.4	0.4	1.7	0.4 to 2.9	0.008***

***p<0.01,

**p<0.05.

The impact of IMNCS on infant and child health indicators using difference-in-difference estimators is shown in Table 4. Knowledge on LBW baby management were higher both in intervention and comparison district simultaneously so the DID estimator was not significant but mothers’ knowledge about the initiation of breastfeeding increased significantly in intervention districts (14.6%, P<0.001) and, as a result, the practice of initiating breastfeeding within an hour of birth also increased significantly (8.3%, P<0.001). Due to the increase in birth asphyxia prevalence in the intervention areas (9.2% to 12.8%), the care provided by trained providers increased significantly in the intervention group compared to the comparison group (4.6%, P<0.01). Complete vaccination of under-five year-old children increased in both the intervention and comparison districts over the time period (negative difference-in-difference estimator -4.5%, P<0.1). Reported prevalence of diarrhea 3 months preceding the survey and other illness decreased in intervention districts whereas diarrhea prevalence increased slightly in comparison districts.

**Table 4:** DID analysis of the impact of the IMNCS intervention on infant and child health indicators.

Outcomes	Intervention		Comparison		DID estimate	95% CI	P value
	Pre	Post	Pre	Post			
**Mother with live children less than one year old or whose child less than one died in past year (n)**	**2092**	**1023**	**1026**	**504**			
Medium knowledge (3–4) on essential newborn care actions (%)	44.5	26.5	45.4	35.5	-6.9	-14.5 to 0.6	0.073*
Knowledge on LBW baby management (frequent breastfeeding and proper wrapping at a time) (%)	44.1	45.6	40.5	41.7	0.6	-7.9 to 9.0	0.897
Knowledge on initiation of breast feeding within 1 hour of birth (%)	69.7	93.2	81.6	89.9	14.6	9.4 to 19.9	0.000***
Received all ENC^1^ (%)	50.6	64.5	51.0	57.7	6.2	-1.4 to 13.8	0.112
Initiation of breastfeeding within 1 hour of birth (%)	77.7	89.5	83.2	86.1	8.3	2.6 to 13.9	0.004***
Having birth asphyxia (%)	9.2	12.8	9.7	9.9	3.3	-1.2 to 7.4	0.150
Care provided by trained care provider during birth asphyxia (%)	3.9	10.5	4.5	6.3	4.6	1.4 to 8.0	0.008***
Prevalence of Neonatal sepsis^1^ (%)	18.9	15.9	21.5	14.5	3.9	-1.9 to 9.7	0.183
Neonatal sepsis managed by medically trained provider (%)	8.1	17.8	15.2	22.2	2.5	-3.4 to 8.4	0.411
**Women who have child under five (n)**	**2400**	**1200**	**1200**	**600**			
Child with complete vaccination^1^ (%)	83.1	84.1	83.4	88.5	-4.5	-9.2 to 0.2	0.058*
Prevalence of ARI (%)	11.1	11.8	15.8	13.5	3.4	-1.5 to 8.2	0.173
ARI managed by medically trained provider (%)	11.4	13.5	14.5	21.7	-5.1	-10.3 to 0.3	0.051*
Reported diarrhea 3 months preceding the survey (%)	13.0	12.4	9.6	9.7	0.7	-3.3 to 4.7	0.733
Reported other illness^1^ 3 months preceding the survey (%)	36.8	27.7	39.2	23.0	7.2	0.5 to 13.8	0.034**

***p<0.01,

**p<0.05,

*p<0.1.

## Discussion

We found the IMNCS intervention resulted in significantly improved most of the mothers, infant and child health indicators compare to their counterpart. Compared to women in comparison areas, the women receiving the improving maternal, neonatal and child survival intervention had improved knowledge of the importance of antenatal care, postnatal care, and hygienic practices during the antenatal period and during delivery and the importance of early initiation of exclusive breastfeeding. These findings are consistent with previous results [18–24]. Furthermore, we observed a significant increase in the use of modern family planning (FP) methods in the IMNCS intervention areas compared to comparison areas of BRAC EHC programme. Adolescent motherhood increased in all improving maternal, neonatal and child survival intervention districts over the five-year period. Therefore, this earlier pregnancy (under the age of 18) might increase the risk of maternal mortality compared to pregnancies occurring later in life(over the ages of 35), consistent with previous studies[25, 26].In addition, it has also been suggested that higher parity increases the risk of maternal mortality[27, 28].Pregnancy can lead to complications in young women due to relatively underdeveloped pelvises being unable to accommodate birth. Some family planning programs have emphasized serving such high-risk women, particularly multiparous women. Many women resort to termination of pregnancies to prevent unintended births [29], which frequently remains illegal in many low-income countries. Therefore, appropriate family planning should be encouraged in women in developing countries, especially younger women, to help avoid possible maternal deaths due to complications arising from unwanted pregnancies and unsafe terminations. Educating young women that family planning is a safe and plausible way to prevent maternal death is vital [30].

Previous studies have documented an association between maternal morbidity and mortality and lack of antenatal care [31, 32].Proper antenatal care is considered a good method to reduce the risk of pregnancy-related morbidity and mortality [33–36]. Antenatal care is particularly relevant in Bangladesh, where maternal mortality is still high[37, 38].Our results revealed that significantly more mothers received at least four antenatal care sessions from trained providers in the intervention group compared to mothers in comparison districts. This would mean that these women were more orientated with the potential complication that might occur during, and after their pregnancy, and be ready to combat those challenges. There was also significant progress in both intervention and comparison areas with respect to major maternal health indicators such as delivery by trained birth attendants, pre- and postnatal complications, and onward referral for complicated cases. Pregnancies were identified by community health workers and entered into the database. The community health workers then followed up those mothers under close supervision by afield manager. Previous research has suggested that close follow-up could increase the chances of individuals accessing health systems [39–44].

Of the infant and child health indicators, there was a significant reduction in knowledge about essential newborn care in intervention mothers compared to comparisons; the reasons for this are unclear. One potential reason could be in the intervention areas, mothers were not aware about the term ‘new born care’, but they understand the care required for their new born baby. However, there was no significant difference in knowledge about the management of low-birth weight babies in both groups, although there were significant improvements in both knowledge and practice about initiating breastfeeding within one hour of birth in the intervention group. This may be due to the intensive messages given about exclusive breastfeeding and early initiation that were repeated in peer counseling at women's group meetings in the intervention group [33, 45–50]. These strategies are used at all levels of communication, and include reminders of the benefits to the child. Societal support of breastfeeding is also promoted during both community and in-person counseling, especially since the actions of family members can either build or challenge a mother's confidence and her ability to adopt new practices. Participation in women's groups during courtyard sessions was open to community members to foster familial support in intervention districts. It has been shown that early breastfeeding for six months provides the foundation for optimal growth and protection against common childhood illness 51. Breastfeeding has a direct link with child development and nutritional status and to ensure a longer duration of early breastfeeding, mothers’ understanding of its benefits needs to be improved. Although the prevalence of birth asphyxia was non-significantly higher in the intervention group than comparisons, the significant number of birth asphyxia cases was managed by trained providers in the intervention area.

The study has captured improvements in certain maternal and neonatal health indicators and identified a number of gaps and challenges with programmatic implications. Nevertheless, there are a few limitations. The quasi-experimental nature of this study is limited in capturing changes in maternal, neonate and child health status across the intervention and comparison districts with the implementation (or not) of the BRAC IMNCS program. One of the limitation of this study was not assigning the intervention and comparison areas randomly, however, the participants were randomly selected from each intervention and comparison areas in order to minimize the selection bias. As this is quasi-experimental study design and it may not convincingly establish the causal link between the intervention and outcome, thus results of this study would require cautious interpretation. In end line we undertook both panel and non-panel data to show the impact of the program indicators. Our limitation also considered half of the sample size of end line data because Difference-in Difference (DiD) only focused on panel. This study was aimed to measure the effect of IMNCS intervention compare to their comparison group. Although the findings associated with the effects of BRAC’s IMNCS are not based on experimental evidence, we are optimistic about the ability of our before-and-after comparison design demonstrates the attribution of the IMNCS intervention to the outcome. However, a randomized control design of such intervention would elucidate further attribution to the outcome. The study assumes that the comparison districts have similar maternal and child health services available via Ministry of Health facilities and non-government organization. However, participants were randomized from sub-district to household level in each district, allowing broader generalization of the results for rural populations in northern and central Bangladesh. Structured face-to-face interviews with pre-tested questionnaires were used to gather information from respondents; reporting bias can occur especially with retrospective reporting, such as of terminations and the details of menstrual regulation. To minimize recall bias, experienced interviewers were used and standardized training about questionnaire and probing techniques was given. Labor, birth, and the immediate postnatal period are the most critical periods for newborn and maternal survival [33, 48].Failure to provide care during these critical periods may lead to detrimental effects on the survival and future health of both mother and baby. There are quite a large number of organization have been working on maternal, neonate and child health issue in Bangladesh, however, a large variation can be seen on the objective of the program or the scope of work. Some work on maternal health, some other work on child health, and many more. A few approaches are comprehensive. Our study suggested an indication that an integrated approach is needed to achieve further reductions in morbidity and mortality. We anticipate that this study would help policy maker to encourage stakeholders, government and international non-governmental organizations (NGO) and other departments to adopt comprehensive approach to deal with the maternal and child health challenges in Bangladesh.

## Conclusion

The study suggests that medically trained health care provider assisted community based public health intervention increases antenatal and postnatal visit, correspondingly decreases ante-partum and intra-partum complications of the mother. However, all major birth plans i.e. delivery place, birth attendant, and saved money does not influence by them. Management of low birth weight and complete vaccination does not effect by the regular visit of medically trained health care providers nevertheless it increases mothers’ knowledge on breastfeeding initiation.

## Supporting Information

S1 Table This file contains the raw data used in modeling, model code, and supplementary results with maternal health. (DOCX)
