# Supplementary material for: A Controlled Before-and-After Perspective on the Improving Maternal, Neonatal, and Child Survival Program in Rural Bangladesh: An Impact Analysis
Source: PLoS One. 2016 Sep 1;11(9):e0161647. doi: 10.1371/journal.pone.0161647 (PMC5008808; doi:10.1371/journal.pone.0161647)
Supplement: S1 Table — (DOCX) [file pone.0161647.s001.docx]

Suporting Information

### S1 Table. This file contains the raw data used in modeling, model code, and supplementary results with maternal health.

Output of Table3
